# The Relationship Among Physical Activity, Generalized Anxiety Disorder, and Suicidal Risk in South Korean Adolescents: Including Individual Characteristics

**DOI:** 10.3390/healthcare13172168

**Published:** 2025-08-30

**Authors:** Jae-Ahm Park

**Affiliations:** Department of Sports and Leisure Studies, College of Humanities, Daegu University, 201 Daegudae-ro, Gyeongsan-si 38453, Republic of Korea; japark@daegu.ac.kr; Tel.: +82-53-850-6087

**Keywords:** physical activity, generalized anxiety disorder, suicidal risk

## Abstract

**Objectives:** This study explored the relationship between physical activity, generalized anxiety disorder, and suicidal risk among South Korean adolescents, considering individual characteristics. **Methods**: The study analyzed raw data from the 2023 Youth Health Behavior Survey conducted by the Korea Disease Control and Prevention Agency. A total of 52,880 adolescents’ data (weighted to 2,581,964) were analyzed using a complex sample design. **Results**: Physical activity level had a negative effect on anxiety. Anxiety had a positive effect on suicidal risk, including thoughts, plans, and attempts. Physical activity level indirectly affected suicidal risk negatively by mediating anxiety. Individual characteristics such as gender, alcohol consumption, smoking, drug use, and household economic status also influenced suicidal risk. **Conclusions**: This study emphasizes that effectively reducing adolescent suicide rates requires a multifaceted approach. Such an approach should include promoting physical activity, addressing mental health challenges like anxiety, and providing tailored support for vulnerable populations.

## 1. Introduction

In South Korea, adolescent suicide has become a growing societal concern. In 2022, it was the leading cause of death among adolescents (10.8%), followed by safety-related accidents (3.9%) and malignant neoplasms (cancer) (2.5%). Although safety-related accidents were the primary cause of adolescent deaths in 2010, suicide has held the top position for 11 consecutive years since 2011 [1]. A research team from Hallym University in Korea conducted psychological autopsies on 89 middle and high school students who had died by suicide, using data from the Ministry of Education of South Korea [2]. The autopsies revealed that, among the 75 students for whom the concerns leading to suicide were identifiable, many struggled with academic performance (26.8%), depression (21.1%), family conflict (18.3%), peer conflict (7.7%), and relationship issues (6.3%).

Notably, while concerns about academic performance were the most common issue, 18.7% of these students were actually high achievers [2]. Furthermore, many of the students who died by suicide lived in stable environments, without notable physical health problems, economic challenges, or housing issues. Instead, their families’ economic status was often classified as “high” (10.1%) or “middle” (65.2%). This suggests that these adolescents perceived themselves and their circumstances more pessimistically than the actual situation warranted. Such negative psychological perspectives may have exacerbated other mental health factors, such as anxiety and depression, ultimately contributing to the decision to take their own lives.

Similar patterns were observed in data from the Korea Youth Policy Institute [3]. A survey of 5937 adolescents found that 17.4% exhibited symptoms of depression, and 13.0% exhibited symptoms of anxiety, with 16.4% classified as being at high risk for suicide. The study also revealed that female students experienced higher levels of mental health issues, including depression, anxiety, and suicide risk, compared to male students, with severity increasing across higher school grades.

Furthermore, previous research has demonstrated a link between anxiety and suicidal ideation among adolescents [4,5,6]. An analysis of 391 adolescents indicated that elevated levels of depression and anxiety were statistically significant predictors of suicidal ideation, with higher levels correlating with more frequent suicidal thoughts [4]. Another study by Kim and Jeon [5] confirmed that depression and anxiety levels in both male and female students had a statistically significant impact on suicidal ideation. Song et al. [6] further demonstrated that individuals with both depression and anxiety had significantly higher impulsivity and suicidal ideation scores compared to the control group, with depression, anxiety, and their combined scores all significantly correlated with suicidal ideation. Additionally, elevated levels of suicidal thoughts and planning were found to increase the likelihood of actual suicide attempts [7]. When suicidal ideation is combined with other negative psychological states, such as major depressive disorder or bipolar disorder, the risk of suicide attempts further intensifies. Therefore, reducing suicidal ideation and planning, as well as maintaining a positive psychological state, is crucial for lowering actual suicide attempts.

Physical activity has been identified as an effective intervention for improving negative psychological states, such as anxiety and depression. A study involving 11,110 adolescents found that more frequent physical activity and participation in sports were independently associated with greater well-being and reduced anxiety and depressive symptoms in both sexes [8]. Another study by Broocks and colleagues [9] compared the effects of aerobic exercise, antidepressant medication, and placebo on patients with panic disorder over eight weeks. The findings revealed that both exercise and medication significantly improved outcomes compared to placebo, with medication being slightly more effective. A systematic review confirmed the beneficial effects of physical activity on reducing anxiety and depression, with these effects persisting for at least 12 months [10].

In addition to mental health, individual factors such as gender, alcohol consumption, smoking, and family economic status have been found to influence the risk of suicide. For instance, female students are 1.77 times more likely to have suicidal thoughts compared to male students [11]. A longitudinal analysis of three years of data from second-year middle school students indicated that drinking behavior is positively correlated with levels of suicidal thoughts [12]. Students who consumed alcohol were 1.35 times more likely to have suicidal thoughts than non-drinkers [11]. Current smokers were also at higher risk, with those smoking 20 or more cigarettes per day being 1.81 times more likely to experience suicidal thoughts than those who smoked 1 to 9 cigarettes per day. Drug use has also been identified as a significant factor affecting suicidal thoughts, plans, and attempts among adolescents [13]. Moreover, students who reported their family’s economic condition as “low” were 1.23 times more likely to have suicidal thoughts compared to those who reported it as “high” [11].

**Figure 1:**
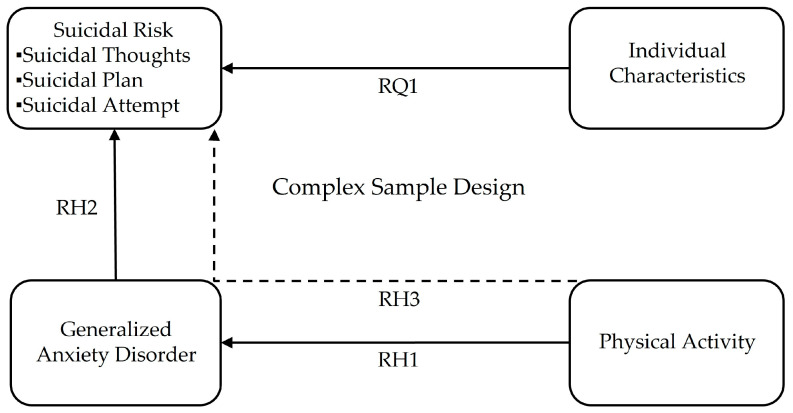
Research model. Solid lines indicate direct effect. Dotted lines indicate indirect effect.

Therefore, the purpose of this study is to investigate how physical activity influences suicidal ideation by reducing anxiety, providing evidence that physical activity can serve as an effective strategy in policies aimed at lowering adolescent suicide rates. Additionally, the study aims to analyze individual characteristics that directly affect suicide risk, offering comprehensive data to support adolescent suicide prevention efforts. This study will apply a complex sample design to provide analytical data representative of the population of South Korea. To achieve this goal, research hypotheses, a research question, and research model (Figure 1) are established based on the studies reviewed above.

**Research** **Hypothesis** **1** **(H1).** *Physical activity level will have a negative effect on anxiety.*

**Research** **Hypothesis** **2** **(H2).** *Anxiety will have a positive effect on suicidal risk (thoughts, plan, attempt).*

**Research** **Hypothesis** **3** **(H3).** *Physical activity level will have an indirect negative effect on suicidal risk (thoughts, plan, attempt) mediated by the anxiety.*

**Research** **Question** **1** **(RQ1).** *What individual characteristics affect suicidal risk (thoughts, plans, attempts)?*

## 2. Materials and Methods

### 2.1. Samples

The present study analyzed raw data from the 2023 Youth Health Behavior Survey conducted by the Korea Disease Control and Prevention Agency [14]. This survey, conducted annually since 2005, is an anonymous, self-administered online questionnaire targeting middle and high school students. It aims to assess the current status and trends of health behaviors among adolescents in Korea. The 19th survey (2023) was conducted across 799 schools, consisting of 399 middle schools and 400 high schools with a total of 52,880 participating students. To ensure that the collected data accurately represents the population of South Korea, a complex sample design—including weights, stratification, and clusters—was applied. Consequently, the final dataset used for analysis in this study consisted of 2,581,964 individuals (Table 1). The weight variable was calculated by the Korea Disease Control and Prevention Agency [14] and included in the raw data.

### 2.2. Complex Sample Design

The sample for the 2023 Youth Health Behavior Survey was extracted using a complex sampling design rather than simple random sampling; therefore, weights, stratification, and cluster variables were considered when estimating means and variances [14]. The final adjusted weights represent 28,401 middle school respondents and 24,479 high school respondents, corresponding to 1,314,377 middle school students and 1,267,587 high school students nationwide as of April 2023. Detailed information on the complex sample design is available in the “Guidelines for Using the 19th (2023) Youth Health Behavior Survey Raw Data”, published by the Korea Disease Control and Prevention Agency [14].

### 2.3. Procedure

Prior to conducting the survey, sample schools were selected, and survey support teachers for these schools were appointed and trained. On the day of the survey, the survey support teacher assigned one internet-enabled mobile device to each student in the sample classes. The survey support teacher distributed a student guide sheet to each student and explained the purpose and process of participating in the survey. Students accessed the survey system using the participation number printed on their guide sheets and completed the survey during class time under the supervision of the survey support teacher. Upon completion, the survey support teacher registered the survey conditions (such as the number of students who completed or did not complete the survey) in the survey system.

### 2.4. Instruments

The 2023 Youth Health Behavior Survey includes 88 questions related to adolescents’ health behaviors and individual characteristics. For the purpose of this study, specific questions regarding adolescents’ levels of physical activity, generalized anxiety disorder, suicidal risk (thoughts, suicide planning, and suicide attempts), individual characteristics were selectively used.

The physical activity section consists of three questions: one asking about the number of days per week with at least 60 min of physical activity, another asking about the number of days with vigorous physical activity, and a third question asking about the number of days with muscle-strengthening exercises. Each question was scored on an 8-point scale (1 = 0 days, 2 = 1 day, 3 = 2 days, 4 = 3 days, 5 = 4 days, 6 = 5 days, 7 = 6 days, 8 = 7 days). A higher score indicates a higher level of physical activity. Based on the average score of 3.023, participants were classified into a low exercise group if their score was below the average, and a high exercise group if their score was above the average.

Generalized anxiety disorder was assessed with 8 items. Example items include: “Feeling nervous, anxious, or on edge,” “Becoming easily annoyed or irritable,” and “Feeling afraid as if something awful might happen”. Each question was scored on a 4-point scale (1 = Not at all disturbed, 2 = Disturbed for several days, 3 = Disturbed for more than 7 days, 4 = Disturbed nearly every day). A higher score indicates a higher level of anxiety. Based on the average score of 1.603, participants were classified into a low anxiety group if their score was below the average, and a high anxiety group if their score was above the average.

Suicidal risk was assessed in three areas: suicidal thoughts, planning, and attempts, with each area assessed by a single question. The questions were: “Have you seriously thought about suicide in the past 12 months?”, “Have you made a specific plan to commit suicide in the past 12 months?”, and “Have you attempted suicide in the past 12 months?”. The response options were (1 = No; 2 = Yes). For the analysis, all responses were re-coded as dummy variables (0 = No, 1 = Yes).

Individual characteristics included gender, alcohol consumption experience, smoking experience, drug use, and household economic status. Alcohol consumption was evaluated by asking whether participants had ever consumed alcohol in their lifetime, with responses coded as 1 for “No” and 2 for “Yes”. Smoking was examined by asking if participants had ever smoked, with responses coded in the same way. Drug use was measured with the question: “Have you ever habitually or intentionally used drugs (e.g., tranquilizers, stimulants, sleeping pills, appetite suppressants, narcotic painkillers) or inhaled substances like glue, marijuana, cocaine, butane gas, etc.?”. Responses were coded as 1 for “No” and 2 for “Yes’. Household economic status was assessed by the question, “How would you describe your household’s economic status?”, with responses categorized as 1 for “Very high”, 2 for “High”, 3 for “Average”, 4 for “Low”, and 5 for “Very low”. Responses were then recoded, where responses of “Low” or “Very low” were grouped as 1, representing the “Low household economic group”, and responses of “Very high”, “High”, or “Average” were grouped as 2, representing the “High household economic group”.

### 2.5. Data Analysis

Statistical analyses were conducted using SPSS 20.0 and SAS 9.4. Descriptive statistics and chi-square tests for complex sample designs (including weight, strata, and cluster variables) were performed using SPSS 20.0. Additionally, accounting for the complex sample design, confirmatory factor analysis, simple regression, logistic regression, and bootstrapping were carried out using SAS 9.4. For simple regression, the mean scores of physical activity and generalized anxiety disorder were used. In the case of logistic regression, the analysis included the mean score of generalized anxiety disorder and dummy variables for suicidal thoughts, plans, and attempts (0 = No, 1 = Yes).

## 3. Results

### 3.1. Confirmatory Factor Aalaysis

Analyzing the measurement model’s fit yielded the following results: [*χ*^2^ = 10,612.7284, *df* = 32, *p* < 0.001], CFI = 0.961, SRMR = 0.029, and RMSEA = 0.07. These findings suggest that the model aligns well with the general criteria for acceptable fit indices [15]. In terms of reliability, all factors demonstrated satisfactory levels, with Cronbach’s alpha values exceeding 0.70 [16]. Concerning convergent validity, all factors met the required standards, with composite reliability (CR) values surpassing 0.70 and average variance extracted (AVE) values exceeding 0.50 [16]. For discriminant validity, the AVE of each factor was greater than the squared correlations with all corresponding factors, confirming discriminant validity [17]. The squared correlation between physical activity and anxiety was found to be 0.009. Detailed results are presented in Table 2 and Table 3.

### 3.2. The Relationship Among Physical Activity, Anxiety and Suicidal Risk

#### 3.2.1. The Effect of Physical Activity Level on Anxiety (RH1)

The simple regression analysis, incorporating a complex sample design, was conducted to examine the effect of physical activity level on anxiety (Table 3). The results of the regression model indicated that the model was statistically significant [*F*(1, 700) = 298.10, *p* < 0.001]. The coefficient for physical activity level was negative and significant [*β* = −0.031, *p* < 0.001], indicating that as physical activity level increases, anxiety decreases. Specifically, a one-unit increase in the mean physical activity score is associated with a 0.031-unit decrease in the mean anxiety score. However, the *R*^2^ value indicates that physical activity level explained approximately 0.71% of the variance in anxiety. Although statistically significant, the small *R*^2^ value suggests that the relationship between physical activity level and anxiety is small, and other variables may contribute to anxiety levels. On the other hand, in a complex sample design, the standard errors are estimated in a way that accounts for the survey’s stratification, clustering, and weighting. This method can influence the test’s sensitivity and significance. Because complex survey designs aim to accurately reflect the population structure, the findings are more generalizable even when the effect size is small.

#### 3.2.2. The Effect of Anxiety on Suicidal Risk (RH2)

A logistic regression with a complex sample design was used to examine the effect of anxiety on suicidal risk (Table 4). First, anxiety had a significant positive effect on suicidal thoughts (*β* = 1.528, *p* < 0.001). The McFadden’s Pseudo *R*^2^ of 0.182 suggests a reasonable model fit. The odds ratio indicates that a one-unit increase in the mean anxiety score is associated with a 4.607-fold increase in the odds of having suicidal thoughts (1 = Yes).

Second, anxiety demonstrated a significant positive effect on suicidal planning (*β* = 1.341, *p* < 0.001). The McFadden’s Pseudo *R*^2^ of 0.149 suggests a reasonable model fit. The odds ratio indicates that a one-unit increase in the mean anxiety score is associated with a 3.822-fold increase in the odds of having a suicidal plan (1 = Yes).

Third, anxiety had a significant positive effect on suicidal attempts (*β* = 1.252, *p* < 0.001). The McFadden’s Pseudo *R*^2^ of 0.125 indicates a reasonable model fit. The odds ratio shows that a one-unit increase in the mean anxiety score is associated with a 3.50-fold increase in the odds of a suicidal attempt (1 = Yes).

#### 3.2.3. Indirect Effect of Physical Activity on Suicidal Risk Mediated by Anxiety (RH3)

The indirect effect of physical activity on suicidal risk, mediated by anxiety, was calculated by multiplying the coefficients from two paths: the effect of physical activity level on anxiety (simple regression) and the effect of anxiety on suicidal risk, which includes suicidal thoughts, plan, and attempts (logistic regression). Bootstrapping with 5000 bootstrap samples and a 95% confidence interval indicated that the indirect effects of physical activity on suicidal risk, mediated by anxiety, were statistically significant, with a *p*-value of less than 0.001. Detailed results are presented in Table 5.

### 3.3. Differences of Suicidal Risk by Individual Characteristics (RQ1)

The results of the complex sample design chi-square analysis revealed statistically significant differences in suicidal thoughts, plans, and attempts based on gender, alcohol consumption, smoking experience, drug use, and household economic status. Detailed results are presented in Table 6.

## 4. Discussion

This study explored the relationship among physical activity, generalized anxiety disorder, and suicidal risk among South Korean adolescents, considering individual characteristics such as gender, alcohol consumption, smoking, drug use, and household economic status. The findings provide valuable insights into the factors contributing to suicidal risk and highlight the role of physical activity in reducing anxiety and, consequently, suicidal risk. The analysis confirmed that higher levels of physical activity are associated with lower levels of generalized anxiety disorder, consistent with prior research suggesting that physical activity effectively improves mental health outcomes [8,9,10]. McMahon et al. [8] examined a sample of 11,110 adolescents from ten European countries. Their multi-level mixed-effects model revealed that increased frequency of physical activity and participation in sports were independently associated with enhanced well-being and lower levels of anxiety and depressive symptoms in both boys and girls. Knowles et al. [10] conducted a systematic review of published studies, finding that physical activity was negatively associated with depression in 61.2% (41 of 67) of the reviewed papers and with anxiety in 57.1% (20 of 35).

Additionally, this study found that anxiety significantly influences suicidal ideation, planning, and attempts, which aligns with previous research. Adolescents with higher anxiety levels were more likely to experience suicidal thoughts, formulate plans, or make attempts, underscoring the critical role of anxiety in adolescent mental health [4,5,6]. Previous studies [4,5,6] provide valuable context for interpreting the findings of this study, as they also focus on the Korean youth population. An interesting aspect of these studies [4,5,6] is their inclusion of both depression and anxiety as variables for predicting suicidal risk among adolescents. Previous research suggests that a broader range of factors, beyond anxiety alone, may influence suicidal risk and offer stronger predictive power when considered together. The findings of this study reaffirm that anxiety is a significant contributor to the increased risk of suicide in adolescents. Therefore, reducing anxiety through interventions such as physical activity may help lower suicide risk in this population.

The key finding of this study is that increasing physical activity can indirectly lower suicidal risk by reducing anxiety. However, the average weekly physical activity time for Korean teenagers in 2023 was only 4.2 h [1]. Adolescents aged 9 to 12 were the most active, averaging 4.6 h of physical activity per week, while those aged 13 to 18 experienced a decrease to an average of 3.8 h per week. This decline may be attributed to reduced leisure time, as adolescents increasingly focus on private education after school hours. In 2023, the participation rate in private education among elementary, middle, and high school students reached 78.5%, an increase from the previous year [1]. In contrast, the average weekly participation time decreased slightly to 7.3 h. In South Korea, especially as the college entrance exam period approaches, school hours for physical education are often reduced in favor of core subjects like Korean, English, and mathematics, which are emphasized in the exams. The World Health Organization (WHO), American Heart Association (AHA), and U.S. Centers for Disease Control and Prevention (CDC) all recommend engaging in at least 150 min of moderate-intensity physical activity per week to enhance cardiovascular health, reduce chronic disease risk, and improve overall physical and mental well-being [18,19,20]. However, only about 25.8% of South Korean high school physical education curricula currently meet these recommended activity levels [21].

Beyond anxiety and physical activity, other individual characteristics such as gender, alcohol consumption, smoking, drug use, and household economic status were also found to impact suicidal risk in this study. These findings support previous research [11,12,13]. Female students were more likely to experience suicidal thoughts than male students, a finding consistent with existing literature on gender differences in mental health [11]. Similarly, adolescents who reported alcohol consumption, smoking, or drug use were more likely to experience suicidal thoughts, plans, and attempts, behaviors commonly linked to poor mental health and increased suicide risk [11,12,13]. Moreover, this study found socioeconomic factors played a significant role in suicidal risk, with students from lower economic backgrounds reporting higher rates of suicidal ideation, plans, and attempts than those from more affluent backgrounds. This underscores the importance of considering socioeconomic disparities when addressing adolescent mental health and suicide prevention.

These findings have several practical implications for policymakers and educators. Promoting physical activity as a regular part of the school curriculum could effectively improve mental health outcomes and reduce anxiety, ultimately lowering suicidal risk among adolescents. Moreover, targeted interventions should be developed for adolescents engaging in risky behaviors such as alcohol consumption, smoking, and drug use, as they are at an increased risk of suicidal ideation. Finally, additional support should be provided to students from lower socioeconomic backgrounds, who may face unique challenges that contribute to mental health struggles. This study provides essential information to help identify adolescents at increased risk of suicide.

Given the limitations of this study, several directions for future research are recommended. First, while the findings indicate that increased physical activity is associated with lower anxiety levels, the small effect size suggests that other factors are also likely to influence anxiety. Future research should explore additional factors that contribute to reducing anxiety among adolescents. Second, a more detailed analysis of individual characteristics should be conducted. For example, further investigation is needed to determine whether the frequency of drinking and smoking correlates with suicide risk, and a more detailed analysis of the frequency and types of drug use would provide meaningful data for suicide prevention efforts. Third, this study only focused on students attending school. Therefore, further research should be conducted to establish suicide prevention measures for adolescents not currently attending school. While students in school may benefit from various suicide risk reduction programs within the institutional framework, out-of-school adolescents might find it more challenging to access such support.

## 5. Conclusions

In conclusion, to effectively reduce adolescent suicide rates, a multifaceted approach is essential. This approach should include promoting physical activity, addressing mental health challenges such as anxiety, and providing tailored support for vulnerable populations. By integrating these strategies into school physical education curricula, mental health policies, and various suicide prevention programs, the risk of suicide among adolescents can be decreased. In particular, the importance of physical activity for adolescents should be recognized in both educational policy and school settings.

## Figures and Tables

**Table 1:** Demographic information (*N* = 2,581,964.000).

Type			Weighted		Unweighted
			*N*	%	*N*
Gender	Male		1,330,425.000	51.5	26,769
	Female		1,251,539.000	48.5	26,111
	Total		2,581,964.000	100.0	52,880
Age	12		125,326.971	4.9	2713
	13		432,816.778	16.8	9354
	14		432,283.001	16.8	9452
	15		448,930.421	17.4	9340
	16		448,526.827	17.4	8777
	17		406,849.354	15.8	7921
	18		283,456.061	11.0	5245
School year	Middle school	1	448,707.000	17.4	9646
		2	423,840.000	16.4	9344
		3	441,830.000	17.1	9411
	High school	1	464,319.000	18.0	9078
		2	411,687.000	15.9	8144
		3	391,581.000	15.2	7257
Suicidal risk	Suicidal thoughts	No	2,234,293.242	86.5	45,749
		Yes	347,670.758	13.5	7131
	Suicidal plan	No	2,446,123.436	94.7	50,089
		Yes	135,840.564	5.3	2791
	Suicidal attempt	No	2,500,146.803	96.8	51,186
		Yes	81,817.197	3.2	1694
Physical activity	Low		1,600,567.807	62.0	32,402
	High		981,396.193	38.0	20,478
Generalized anxiety disorder	Low		1,647,981.754	63.8	33,839
	High		933,982.246	36.2	19,041
Drinking alcohol	No		1,740,109.670	67.4	35,604
	Yes		841,854.330	32.6	17,276
Smoking	No		2,359,566.137	91.4	48,341
	Yes		222,397.863	8.6	4539
Drug use	No		2,539,177.221	98.3	52,041
	Yes		42,786.779	1.7	839
Household economic status	Low		302,431.932	11.7	6484
	High		2,279,221.468	88.3	46,391

**Table 2:** Detailed description of variables (*N* = 2,581,964.000).

Variable		M	S.D.	SRW	α	CR	AVE
Physical activity	Item1	3.19	2.160	0.785	0.815	0.820	0.606
	Item2	3.35	2.084	0.877			
	Item3	2.53	2.090	0.658			
Generalized anxiety disorder	Item1	1.61	0.794	0.818	0.905	0.908	0.588
	Item2	1.64	0.851	0.852			
	Item3	2.00	0.960	0.794			
	Item4	1.51	0.802	0.789			
	Item5	1.31	0.661	0.723			
	Item6	1.76	0.900	0.641			
	Item7	1.38	0.741	0.728			

M = mean, S.D. = standard deviation, SRW = standardized regression weights, α = Cronbach’s alpha, CR = construct reliability, AVE = average variance extracted, Squared Correlation between Physical Activity and Anxiety = 0.0090.

**Table 3:** Complex sample design simple regression analysis for anxiety predicted by physical activity level.

Effect	Estimate (*β*)	S.E.	*t*-Value	*p*-Value
Intercept	1.696	0.007	229.79	<0.001
Physical activity	−0.031	0.002	−17.27	<0.001

**Table 4:** The effect of anxiety on suicidal risk (*N* = 2,581,964).

Independent	Dependent	*β*	S.E.	Odd Ratio (95% CI)
Anxiety	Suicidal thoughts (0 = no, 1 = yes)	1.528 ***	0.021	4.607 (4.417, 4.804)
Model *χ*^2^ = 371,455.6 ***, McFadden’s Pseudo *R*^2^ = 0.182				
**Independent**	**Dependent**	***β***	**S.E.**	**Odd Ratio**
Anxiety	Suicidal plan	1.341 ***	0.026	3.822 (3.633, 4.020)
Model *χ*^2^ = 159,069.36 ***, McFadden’s Pseudo *R*^2^ = 0.149				
**Independent**	**Dependent**	***β***	**S.E.**	**Odd Ratio**
Anxiety	Suicidal attempt	1.252 ***	0.029	3.50 (3.31, 3.70)
Model *χ*^2^ = 90,751.75 ***, McFadden’s Pseudo *R*^2^ = 0.125				

*** = *p* < 0.001.

**Table 5:** Indirect effect of physical activity on suicidal risk mediated by anxiety.

Path	*β*	95% CI	
		Lower Limit	Upper Limit
Physical activity ⟶ anxiety⟶ suicidal thoughts (0 = no, 1 = yes)	−0.0473 ***	−0.0412	−0.0536
Physical activity ⟶ anxiety⟶ suicidal plan (0 = no, 1 = yes)	−0.0415 ***	−0.0359	−0.0471
Physical activity ⟶ anxiety⟶ suicidal attempt (0 = no, 1 = yes)	−0.0389 ***	−0.0337	−0.0442

*** = *p* < 0.001.

**Table 6:** Differences of suicidal risk by individual characteristics.

**Gender**	**Suicidal Thoughts**		**Suicidal Plan**		**Suicidal Attempts**	
	**No**	**Yes**	**No**	**Yes**	**No**	**Yes**
Male	90.2	9.8	96.0	4.0	97.5	2.5
Female	82.6	17.4	93.4	6.6	96.1	3.9
Total	86.5	13.5	94.7	5.3	96.8	3.2
*χ* ^2^	423.071 ***		122.753 ***		65.542 ***	
**Drinking**	**Suicidal Thoughts**		**Suicidal Plan**		**Suicidal Attempts**	
	**No**	**Yes**	**No**	**Yes**	**No**	**Yes**
No	88.6	11.4	95.8	4.2	97.8	2.2
Yes	82.3	17.7	92.4	7.6	94.9	5.1
Total	86.5	13.5	94.7	5.3	96.8	3.2
*χ* ^2^	288.099 ***		221.814 ***		251.060 ***	
**Smoking**	**Suicidal Thoughts**		**Suicidal Plan**		**Suicidal Attempts**	
	**No**	**Yes**	**No**	**Yes**	**No**	**Yes**
No	87.6	12.4	95.4	4.6	97.3	2.7
Yes	75.7	24.3	87.9	12.1	91.4	8.6
Total	86.5	13.5	94.7	5.3	96.8	3.2
*χ* ^2^	331.832		340.867 ***		356.587 ***	
**Drug**	**Suicidal Thoughts**		**Suicidal Plan**		**Suicidal Attempts**	
	**No**	**Yes**	**No**	**Yes**	**No**	**Yes**
No	87.1	12.9	95.3	4.7	97.2	2.8
Yes	50.6	49.4	63.7	36.3	72.8	27.2
Total	86.5	13.5	94.7	5.3	96.8	3.2
*χ* ^2^	719.389 ***		1289.451 ***		1227.526 ***	
**Econ**	**Suicidal Thoughts**		**Suicidal Plan**		**Suicidal Attempts**	
	**No**	**Yes**	**No**	**Yes**	**No**	**Yes**
Low	77.2	22.8	89.8	10.2	93.7	6.3
High	87.8	12.2	95.4	4.6	97.2	2.8
Total	86.5	13.5	94.7	5.3	96.8	3.2
*χ* ^2^	448.764 ***		302.142 ***		186.560 ***	

*** = *p* < 0.001.

## Data Availability

Restrictions apply to the availability of these data. Data were obtained from [Korea Disease Control and Prevention Agency] and are available at [https://www.kdca.go.kr/yhs/ (accessed on 10 August 2025)] with the permission of [Korea Disease Control and Prevention Agency].
